# A Rare Case of Diffuse Neurofibroma of the Scalp With Destructive Lesions Involving the Base of the Skull in a Patient With Neurofibromatosis Type 1

**DOI:** 10.7759/cureus.13930

**Published:** 2021-03-16

**Authors:** Hamza Maqsood, Muhammad Saim, Abdul Sattar Anjum, Shifa Younus

**Affiliations:** 1 Cardiology, Nishtar Medical University, Multan, PAK; 2 Radiology, Nishtar Medical University, Multan, PAK; 3 Medicine, Nishtar Medical University, Multan, PAK

**Keywords:** neurofibroma, neurofibromatosis, osteolytic, skull defects, calvarial

## Abstract

Diffuse neurofibroma is a benign tumor of peripheral nerves. Ten percent of neurofibromatosis type 1 (NF-1) patients can develop diffuse neurofibroma. Here, we report a case of diffuse neurofibroma involving the base of the skull in a 50-years-old patient with NF-1. The patient presented with diffuse involvement of the scalp with soft and mobile masses. Radiological investigations revealed skull bone lesions. Aggressive osteolytic lesions involving the base of the skull were present. Surgical excision with the repairing of the defects was suggested but the patient refused the treatment. The diagnosis of calvarial defects in diffuse neurofibroma is challenging. Early diagnosis can help in better management of the patients.

## Introduction

Neurofibroma is a benign peripheral nerve sheath tumor with different types; localized, plexiform, and diffuse. Diffuse neurofibroma is a unique and uncommon class of neurofibroma [1]. About 10% of patients with neurofibromatosis type 1 (NF-1) have diffuse neurofibroma that involves the head, trunk, and legs. The clinical manifestations appear at a young age and so diagnosed earlier [2].

Over 50% of patients with NF-1 have osseous defects, and among those, the skull bone defects are of rare occurrence. Sphenoid wing dysplasia and orbital defects are the most common calvarial anomalies in patients with NF-1 [3]. So far, no case reporting involvement of base of the skull in a patient of middle age with NF-1 has been documented.

## Case presentation

**Figure 1:**
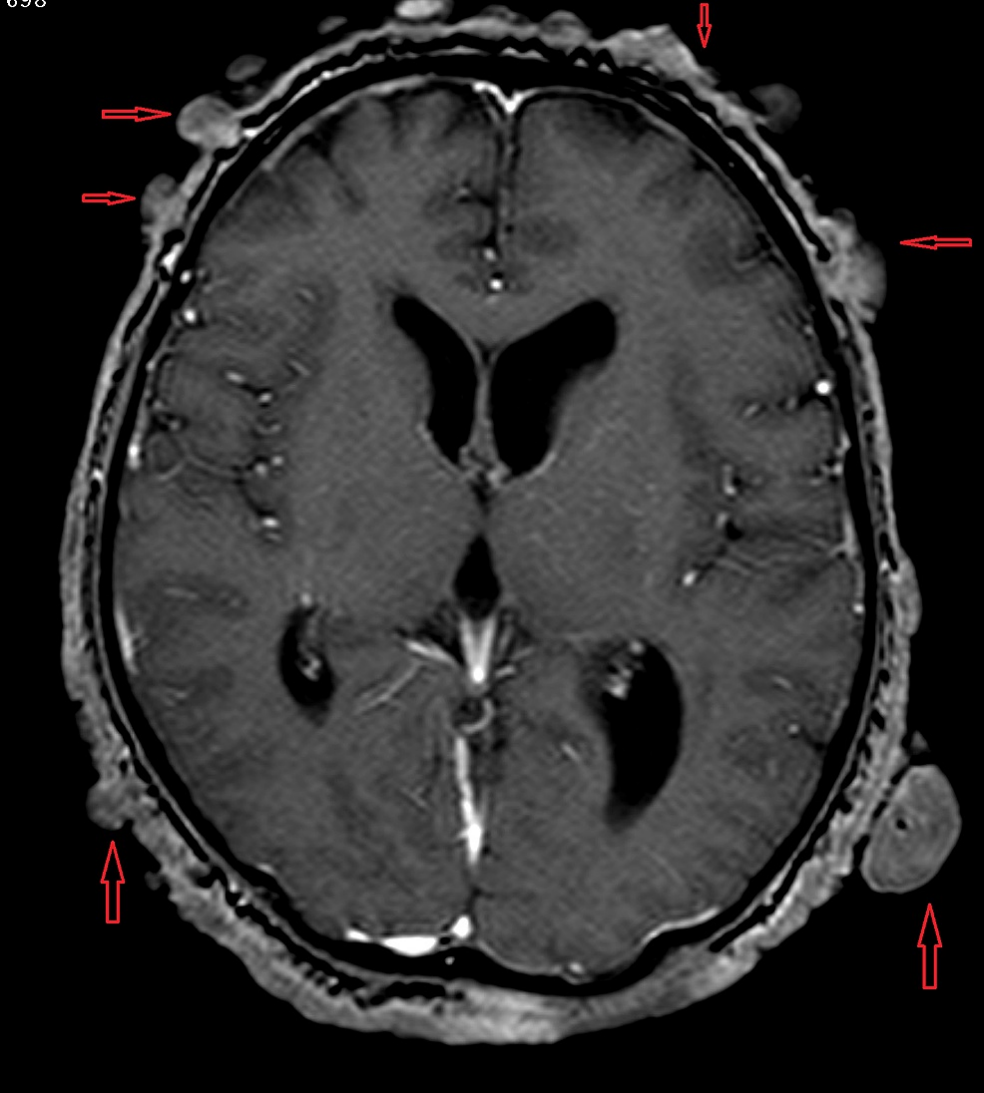
Axial post-contrast computed tomography scan of the head showing multiple growths (red arrows) on the scalp.

**Figure 2:**
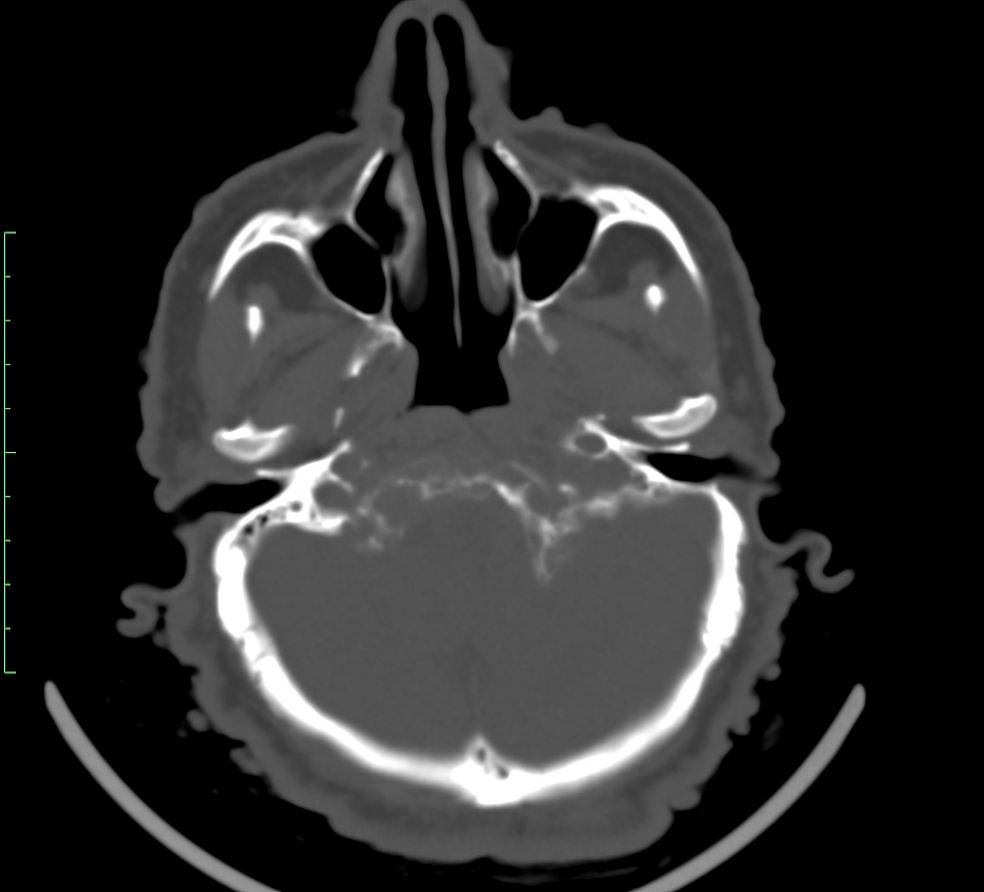
Computed tomography scan showing the aggressive destruction of the bones of the base of the skull.

Here, we report a case of a 50-years-old female who presented in a medical emergency with complaints of altered level of consciousness from six hours and ataxia for three days. The patient was a known case of NF-1 for 35 years. The patient was unmarried and had no pregnancy. There was no history of any other disorder. On general physical examination, we found marked pallor and emaciation. Her scalp examination revealed various slow-growing, mobile, and soft masses present all over the scalp. There were no changes in the bone around the margins of the swollen mass. Diffuse café au lait spots were present all over the body. Relevant neurological and ophthalmological examination was normal. We did a computed tomography (CT) scan of the skull, which showed multiple neurofibromas in the scalp. It also revealed destructive lesions of bones of the skull base and a lytic lesion involving the left frontal bone (Figures 1 and 2).

**Figure 3:**
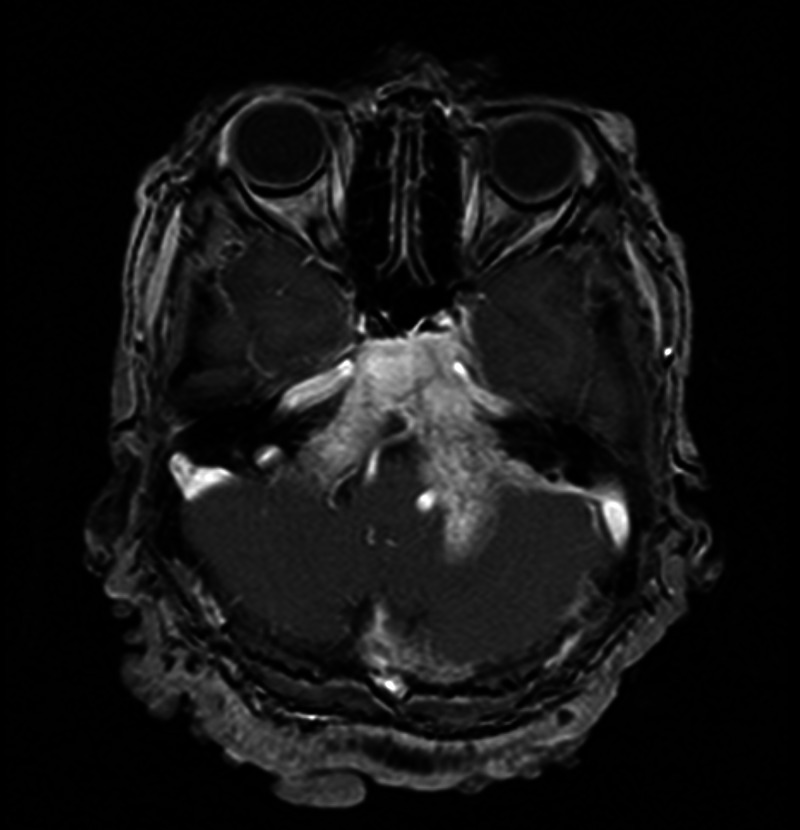
Magnetic resonance imaging scan of the brain showing destructive skull bone lesions along with compressing effect on the pons and the medulla oblongata.

Contrast-enhanced magnetic resonance imaging (MRI) scan of the brain with intravenous gadolinium was done later. It revealed aggressive destructive lesions involving bones of the base of the skull causing compression effect on pons and medulla oblongata (Figure 3).

The case was discussed at the monthly conference of neurologists and neurosurgeons. Surgical removal of neurofibroma along with repairing the base of the skull was suggested. The patient and her attendants refused to undergo surgical management therapy. The patient was managed conservatively and discharged after 14 days. We followed up with the patient after six months. There was no change in the size of the lesion and the severity of symptoms.

## Discussion

Neurofibromas are well-known benign tumors of the peripheral nerve sheath. According to their growth patterns, they may be categorized as local, plexiform, and diffuse types. Diffuse neurofibromas occur most commonly among children and young adults [4]. Diffuse neurofibromas are mostly isolated but in 10% of cases, they are associated with NF-1 [2]. Diffuse neurofibromas are non-encapsulated tumors. They are usually located in the subcutaneous tissue and can infiltrate down to the level of the fascia [4].

Very few cases of diffuse neurofibroma involving the skull are present in the literature. The underlying mechanism causing these lytic bone defects is still unknown. The skull bone defects may be attributed to resorption of bone due to overlying plexiform neurofibroma [3].

Bony lesions can be observed on plain radiographs or CT scans. MRI is done preoperatively to assess the extension of the lesion as it may be close to the dura or can invade the sinuses [1,5].

The management of large neurofibromas involves partial or complete surgical excision. But there no guidelines available for the management of skull bone defects in diffuse neurofibroma. Multiple surgical techniques have been tried but the success rate was under target because of the progressive nature of the disease [6]. A regular follow-up is, therefore, recommended for evaluation of the lesion.

## Conclusions

Diffuse neurofibroma is a benign tumor and lacks the distinctive clinical feature. The diagnosis is very challenging and therefore diffuse neurofibroma should be included in the differential diagnosis of any indurated mass on the scalp. Early diagnosis can help in better management of the patients.
